# Analgesic and potentiated photothermal therapy with ropivacaine-loaded hydrogels

**DOI:** 10.7150/thno.81325

**Published:** 2023-04-09

**Authors:** Jiqian Zhang, Shasha Zhu, Mingxu Zhao, Mengni Zhou, Xiaoling Zhu, Xin Qing, Zhilai Yang, Pengfei Wei, Guoying Zhang, Weiling He, Yongqiang Yu, Xuesheng Liu

**Affiliations:** 1 Department of Anesthesiology, the First Affiliated Hospital of Anhui Medical University, Key Laboratory of Anesthesia and Perioperative Medicine of Anhui Higher Education Institutes, Anhui Medical University, Hefei, 230032, China.; 2 CAS Key Laboratory of Soft Matter Chemistry, Department of Polymer Science and Engineering, School of Chemistry and Materials Science, University of Science and Technology of China, Hefei, 230032, China.; 3 Department of Radiology, The First Affiliated Hospital of Anhui Medical University, Hefei, 230032, China.; 4 Reproductive Medicine Center, Department of Obstetrics and Gynecology, the First Affiliated Hospital of Anhui Medical University, Hefei, 230032, China.; 5 Department of Gastrointestinal Surgery, Xiang'an Hospital of Xiamen University, School of Medicine, Xiamen University, Xiamen, 361000, China.; 6 School of Pharmacy, Shandong Technology Innovation Center of Molecular Targeting and Intelligent Diagnosis and Treatment, Binzhou Medical University, Yantai, 264003, China.

**Keywords:** cancer, cancer-related pain, Pluronic F127, photothermal therapy, local anesthetics

## Abstract

**Rationale:** Tumor ablation can cause severe pain to patients, but there is no satisfactory means of analgesia available. In addition, recurrence of residual tumors due to incomplete ablation threatens patient safety. Photothermal therapy (PTT), a promising approach for tumor ablation, also faces the aforementioned problems. Therefore, developing novel photothermal agents that can efficiently relieve PTT-associated pain and potentiate the PTT efficacy are urgently needed.

**Methods:** The Pluronic F127 hydrogel doped with indocyanine green (ICG) was served as photothermal agent for PTT. Mouse model that inoculation of tumor near the sciatic nerve was constructed to assess the PTT-evoked pain. Subcutaneous and sciatic nerve vicinal tumor-bearing mice were used to test the efficacy of PTT.

**Results:** PTT-evoked pain depends on an increase in tumor temperature and is accompanied by the activation of TRPV1. A simple introduction of local anesthetic (LA) ropivacaine into ICG-loaded hydrogels relieves PTT-induced pain and exerts long-lasting analgesia compared with opioid analgesia. More interestingly, ropivacaine upregulates major histocompatibility complex class I (MHC-I) in tumor cells by impairing autophagy. Therefore, a hydrogel co-doped with ropivacaine, TLR7 agonist imiquimod and ICG was rationally designed. In the hydrogel system, imiquimod primes tumor-specific CD8^+^ T cells through promoting DCs maturation, and ropivacaine facilitates tumor cells recognition by primed CD8^+^ T cells through upregulating MHC-I. Consequently, the hydrogel maximumly increases CD8^+^ T cells infiltration into tumor and potentiates PTT efficacy.

**Conclusion:** This study for the first time provides an LA-dopped photothermal agents for painless PTT and innovatively proposes that a LA can be used as an immunomodulator to potentiate the PTT efficacy.

## Introduction

Ablation therapy is important for the treatment of tumors in clinical settings. Radiofrequency, microwave, and laser-mediated ablation therapy all have been established in clinical practice for the treatment of different types of solid tumors 1. Photothermal therapy (PTT) is another promising tumor ablation method. Over the past few decades, great strides have been made in the development of effective PTT strategies. However, PTT is always accompanied by elevated temperatures 2 and local inflammatory responses 3, which are major causes of pain 4, 5. To avoid severe pain associated with ablation, surgeons usually operate under general anesthesia and intravenous opioid infusion. In addition, intravenous opioid infusion is also required for postoperative analgesia. Whereas, opioid use has many adverse side effects, such as sedation, dizziness, nausea, vomiting, constipation, physical dependence, tolerance, and respiratory depression 6. Additionally, some patients do not achieve satisfactory analgesia after opioid administration. Since PTT also faces the aforementioned problems, developing novel photothermal agents that can efficiently relieve PTT-associated pain without opioid components is urgently needed. Local anesthetics (LAs) have received increasing attention in the management of various types of pain. LAs are a group of pharmacological agents that reversibly block the conduction of impulses in electrically excitable tissues. LAs injection around the nerve trunk, plexus, or ganglion can block impulse conduction and produce anesthesia in the area they innervate through a technique called “nerve block”. Nerve block has proven effective in relieving many types of pain, such as neuropathic 7, inflammatory 8, and spontaneous cancer pain 9, as well as pain caused by heat or mechanical stimulation 10. Therefore, we speculate that introducing LAs into photothermal agents also relieve PTT-induced pain.

However, the high toxicity at high concentrations and short duration of analgesia severely limits the clinical application of LAs. To overcome these shortcomings, Zhao et al. designed polymer-tetrodotoxin (TTX) conjugates to prolong the duration of local anesthesia with minimal toxicity 11. Zhang et al. developed an injectable hydrogel/microsphere composite co-encapsulating bupivacaine and dexmedetomidine for effective sustained analgesia 12. Moreover, some scholars have applied engineered LAs to animal models of pain. For example, Sahadev et al. used liposomes containing saxitoxin (STX) to provide nerve blocks lasting for approximately 1 week from a single injection in a mouse model of spared nerve injury 7. Carolina et al. reported that liposomal ropivacaine can change the pharmacokinetics of ropivacaine and enhance anesthetic duration in mice with incision pain without significant toxicity to local tissues 13. Our previous study indicated that liposomal ropivacaine combined with calorie restriction therapy can relieve spontaneous cancer pain 14.

In addition to causing pain, recurrence due to incomplete tumor ablation is another major challenge of tumor ablation. Interestingly, beyond to relieve pain, LAs also show antitumor potential. Several studies have revealed that LAs can kill tumor cells 15, inhibit tumor metastasis 16, prevent postoperative recurrence 17, 18 and potentiate conventional antitumor treatments 19. In addition, LAs can relieve surgery-induced immune responses and inflammation which promote tumor progression 20. However, whether LAs can directly modulate the immune system is unclear, and studies on the application of LAs in immunotherapy are lacking. Impaired antigen presentation caused by mutations or loss of heterozygosity of major histocompatibility complex class I (MHC-I) is a common mechanism by which tumor cells evade the immune system 21, 22. Interestingly, Keisuke et al. found that inhibition of autophagic degradation upregulates MHC-I in tumor cells and leads to improved antigen presentation, enhanced antitumor T cell responses, and reduced tumor growth in syngeneic host mice 23. Our previous study showed that ropivacaine, a popular LA, impairs autophagy by damaging lysosomal degradation 14. Therefore, ropivacaine may upregulate MHC-I in tumor cells, which in turn promotes the killing of residual tumor cells after ablation to achieve potentiated photothermal therapy.

**Figure 1:**
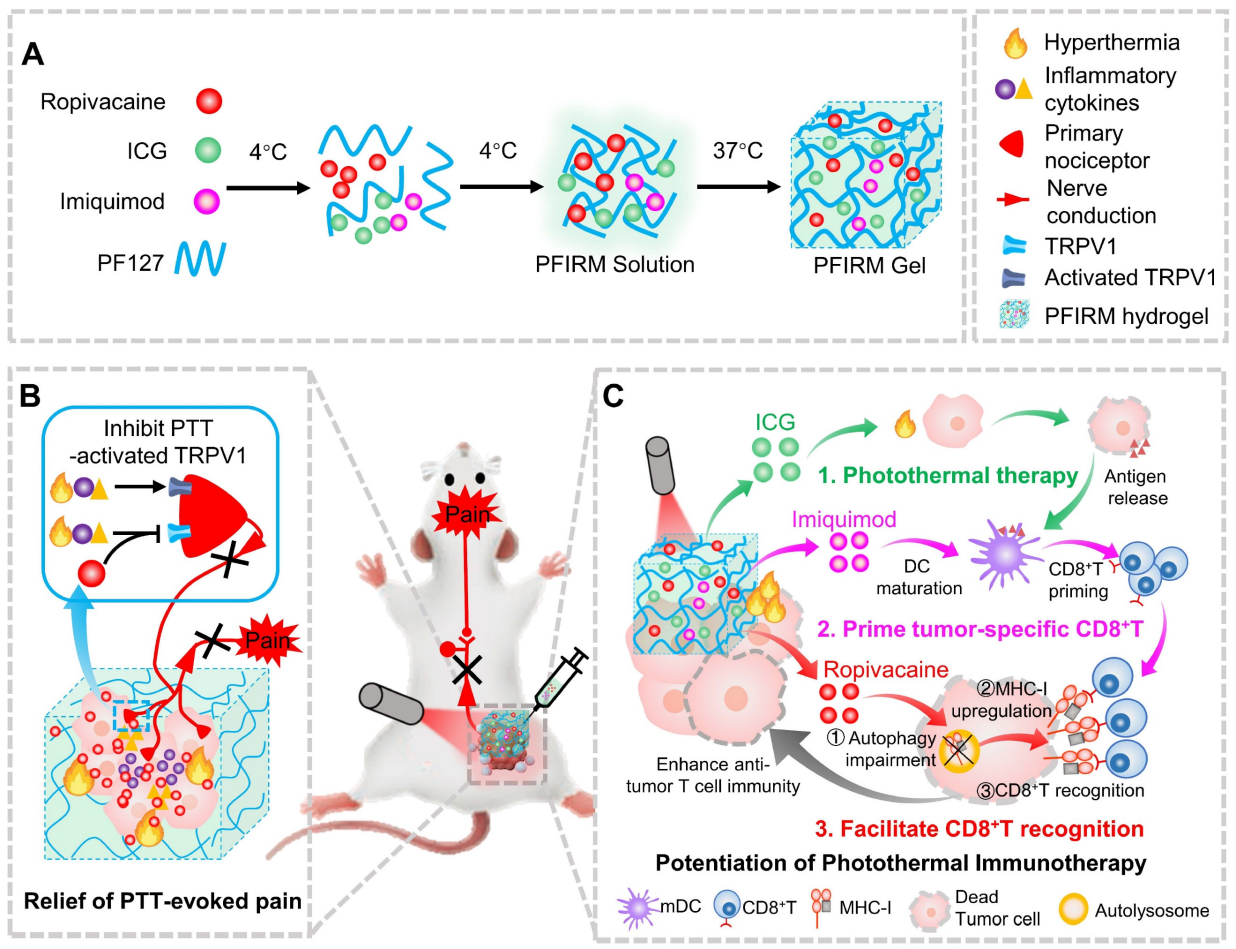
Schematic showing ropivacaine-loaded hydrogels relieve PTT-induced severe pain and simultaneously potentiate photothermal therapy. (A) Preparation of PFIRM hydrogel. (B) PTT raises tumor temperature and promotes the release of inflammatory cytokines that activate TRPV1, resulting in severe pain. Ropivacaine inhibits the activation of TRPV1 and blocks TRPV1-mediated nerve conduction from the peripheral to the central nervous system, resulting in relief of PTT-induced pain. (C) Mechanism of PFIRM-potentiated photothermal therapy. Firstly, ICG-mediated PTT kills tumor cells and promotes their release of tumor-specific antigens; secondly, antigens combined with imiquimod promotes DCs maturation, priming tumor-specific CD8^+^ T cells; finally, ropivacaine upregulates MHC-I in tumor cells, facilitating tumor recognition by primed CD8^+^ T cells.

Pluronic F127 (PF127) is an FDA-approved thermo-responsive biocompatible polymer. In the present study, we prepared an indocyanine green (ICG)-loaded PF127 hydrogel (PFI) as a photothermal agent for PTT in tumor-bearing mice 24. We found that PTT evokes severe acute pain in mice, which correlates with increased tumor temperature and TRPV1 activation. Furthermore, ropivacaine was doped into PFI hydrogel. We found that PTT using ropivacaine-loaded PFI (PFIR) prevents therapy-induced pain and reduces TRPV1 activation. In addition, the ropivacaine was found to impair autophagy and increase MHC-I levels in tumor cells, suggesting its immunotherapy potential, since upregulation of MHC-I is known to promote the recognition of tumor cells by cytotoxic T lymphocytes (CD8^+^ T cells) 23. Imiquimod is an FDA-approved TLR7 agonist that reportedly primes tumor specific CD8^+^ T cells by promoting dendritic cell (DC) maturation 2, 25, 26. Therefore, we rationally design a PF127 hydrogel doped with ICG, ropivacaine and imiquimod (PFIRM). We hypothesized that PFIRM synergistically primes tumor-specific CD8^+^ T cells and facilitates their recognition of tumor cells, potentiating PTT. Indeed, PTT using PFIRM promotes the DCs maturation and upregulates MHC-I expression in tumor cells, resulting in significantly increased CD8^+^ T cells infiltrations into tumors. Consequently, PFIRM enhances PTT efficacy. This study is the first to show that introducing a LA into photothermal agent prevents PTT-induced severe pain, avoiding additional intraoperative and postoperative analgesia. In addition, the present study innovatively proposed that a LA can be used as an immunomodulator to potentiate the efficacy of PTT, providing novel ideas for painless cancer treatment (Figure 1).

## Materials and methods

### Antibodies and agents

Anti-TRPV1 (1:100, ab6166), anti-LC3 (1:1000, ab192890), anti-P62 (1:1000, ab109012), anti-LAMP2 (1:100, ab13524), anti-H2 Db/H2-D1(1:100, ab25244), and Alexa Fluor 568 (1:500), Alexa Fluor 488 (1:500) secondary antibody were purchased from Abcam. Anti-CGRP (1:100, sc-57053) was purchased from Santa cruze. Anti-mouse CD45-PE-cy7 (103114), anti-mouse CD11c-APC-cy7 (117323), anti-mouse CD3-APC-cy7 (100221), anti-mouse CD8a-APC (100711), anti-mouse CD16/32-TruStain FcX™ (101320), anti-mouse CD86-APC (105011) antibodies were purchased from BioLegend. APC anti-mouse H-2 (E-AB-F1216E) and Anti-mouse CD80-FITC (E-AB-F0992C) were purchased from Elabscience. HRP-conjugated anti-rabbit antibody (W4011) and HRP-conjugated anti-mouse antibody (W4021) were purchased from Promega. Enhanced chemiluminescence (ECL) kit was purchased from Biological Industries. One Step TUNEL Apoptosis Assay Kit (Green Fluorescence) (C1088) and Ad-GFP-LC3B (C3006) were purchased from Beyotime. Lyso-Tracker Red (L12492) was purchased from Invitrogen. Imiquimod (HY-B0180A) was purchased from MCE. Indocyanine Green (3599-32-4) was purchased from Aladdin. Morphine (161007-2) was purchased from NORTHEAST PHARM. Ropivacaine (R413090) was purchased from Mackline. Pluronic F127 (P2443) was purchased from Sigma.

### Preparation and characterization of PF127 hydrogels

PF127 hydrogel were prepared by a “cold method” as described in previous reports 27. Briefly, 0.25 g PF127 was mixed with 1mL cold deionized water and stirred gently at 4 ℃ until complete dissolution. For PFI hydrogel preparation, 0.5 mg ICG were added to 1 mL PF127 hydrogel and stirred gently at 4 ℃ to form favored hydrogel. For PFIR hydrogel preparation, 10 mg ropivacaine and 0.5 mg ICG were added to 1 mL PF127 hydrogel and stirred gently at 4 ℃ to form hydrogel. For PFIRM hydrogel preparation, 10mg ropivacaine, 0.5 mg ICG and 0.3 mg imiquimod were added to 1 mL PF127 hydrogel and stirred gently at 4 ℃ to form hydrogel. All gels were kept overnight at 4 °C until a homogenous solution was obtained. The morphology of hydrogels was observed by Carl Zeiss field-emission scanning electron microscope after a lyophilization.

### Rheological analysis of hydrogels

Rheological experiments were performed on Anton Paar (MCR 302) using a parallel plate (plate diameter, 40 mm; gap, 0.5 mm). The storage modulus (G′) and loss modulus (G″) of different combinations were quantified under different conditions. For the temperature-dependent experiments, the heating rate was set at 1.0 °C/min. The sol-gel transition temperature was determined as the intersection point of G′ and G″. On the other hand, frequency-dependent rheological measurements were conducted at 37 °C, with a strain rate of 0.1%. Shear-dependent changes in viscosity was carried out at 37 °C.

### *In vitro* release study

The hydrogel in the glass tube was placed at 37 ℃ to form a gel. 4 mL of 37 °C deionized water was carefully layered on top of the gel layer and the tubes were placed at an orbital shaker set at 37 °C / 70 rpm. At the indicated timepoints (5 min, 2 h, 6 h, 12 h, 24 h, 36 h, 48 h, 60 h and 72 h), 1 mL sample was withdrawn and the volume was replaced with 1 mL deionized 28. After collecting the final timepoint, concentrations of ropivacaine was determined by Micro UV-Vis Spectrophotometer (LIFEREAL, FC-1100). The absorbance of ropivacaine were detected at 262 nm. The cumulative release was calculated according to Eq.: E (%) = (V_E_ ∑_1_^n-1^ C_i_ + V_0_C_n_) / m_0_ × 100; where E (%) is the cumulative release, V_E_ is the withdrawn volume (1 mL), V_0_ is the begin volume (4 mL), C_i_ and C_n_ are the drugs concentrations, *i* and *n* are the sampling times and m_0_ is the total amount of drugs loaded in the hydrogel 28. The release study was performed in triplicate.

### Cell culture, transfection and lysosome staining

Cells were cultured at 37 °C with 5% CO_2_ in Dulbecco's Modified Eagle's Medium (DMEM) supplemented with 10% fetal bovine serum (FBS). For construction of LC3-GFP infected cells, 100,000 cells were seeded in 15 mm dish with glass bottom and cultured in DMEM medium containing FBS. Next day, the medium was changed to fresh DMEM medium containing 5 μL Ad-GFP-LC3B (2×10^5^ pfu), and cells were cultured for another 24 h. Then, the transfected cells were undergone different treatments. For lysosome staining, cells were treated with 75 nM Lyso-Tracker Red for 15 min, followed by two washes with PBS. The cells were visualized using a confocal fluorescence microscope (LSM800, Zeiss). The size of lysosomes was analyzed by Image J software with the ““analyze particles-count/size” tool with default settings”. 4T1 cells were provided by Prof. Longping Wen from the South China University of Technology.

### Immunofluorescence

Cells were fixed using 4% PFA for 10 min, permeabilized with 0.1% Triton X-100 for 10 min, and blocked with 1% FBS for 1 h. Then, cells were incubated with primary antibodies overnight at 4 °C and labeled with secondary antibodies at 37 °C for 1 h. Images were acquired using fluorescence microscopy (IX71, Olympus) or confocal fluorescence microscope (LSM800, Zeiss). For tissue immunofluorescence, mice were anesthetized and perfused with PBS, followed by 4% PFA. Following perfusion, the L3-L5 dorsal root ganglion or tumors were obtained and post-fixed overnight in 4% PFA, then cryoprotected overnight in 30% sucrose in PBS. Frozen tissues were embedded in TissueTek OCT compound, then cut into 10 μm sections. The sections were evaluated for immunofluorescence.

### Western blotting

Cells were lysed with sample buffer and boiled for 10 min. Proteins were separated by sodium dodecylsulfate polyacrylamide gel electrophoresis and were transferred to nitrocellulose membranes. The membranes were incubated with primary antibodies at 4 °C overnight, then with secondary antibodies for 1 h at 37 °C. Membranes were incubated with ECL kit reagents and visualized using a chemiluminescence instrument (Amersham Imager 600, GE Healthcare).

### Animals

Six to eight weeks old female Balb/ c mice, were purchased from the Shanghai SLAC Laboratory animal. All mice were housed in temperature, humidity, and light controlled rooms, with water provided ad libitum. Animal welfare and experimental procedures were performed in accordance with the Ethical Regulations on the Care and Use of Laboratory Animals of Anhui Medical University and were approved by the school committee for animal experiments.

### Behavioral assessment

Mice were anesthetized with isoflurane. One million 4T1 cells in 100 μL of sterile PBS were injected into the muscular tissue in the immediate vicinity of the nerve near the trochanter 24. Ten days after inoculation, the paw was observed to curl up, indicating the successful establishment of a mouse model of spontaneous cancer pain. Tumor-bearing mice were injected with 75 μL PFI or PFIR into tumor near the sciatic nerve. Then tumor near the sciatic nerve was irradiated with 808 nm laser at indicated power (0.15, 0.40 and 1.2 W/ cm^2^) for 5 min. For evaluating the morphine analgesia after PPT with PFI, mice were subcutaneously injected with 5 mg/kg morphine 29 10 min before receiving laser irradiation. The changes of tumor temperature were recorded by Thermal Infrared Imager (H10, HIKVISION) with IVMS-4800 software. The posture of mouse paw was observed and photographed. The mechanical withdrawal threshold was tested using Von Frey anesthesiometer (IITC 2091, IITC Life Science) at indicated timepoints. In brief, mice were placed in plastic cages with wire-net floors. The rigid tip of a 2450 series electronic Von Frey anesthesiometer was placed onto the plantar surface of the hind paw and pressed upward slowly until a withdrawal reflex was observed, and the force that elicited the withdrawal reflex was recorded 24.

### Antitumor assessment

For subcutaneous tumor-bearing mice construction, one million 4T1 cells in 100 μL of sterile PBS were subcutaneously injected into the right flank of mice. When the tumor size approaches about 60 mm^3^, the tumor bearing mice were randomly divided into 5 groups, and were intratumorally injected with 75 μL PBS, PFI, PFIR, PFIM or PFIRM, followed by irradiation with 808 nm laser at 1.2 W/cm^2^ for 5 min. PTT were performed every other day for 3 times. Tumor sizes were measured every 2 days. Tumor volume was calculated using the following formula: length × width^2^ / 2 = tumor volume (mm^3^) 30. When the tumor size neared 2000 mm^3^ 31, all mice were sacrificed and the excised tumors were performed for flow cytometry analysis, immunofluorescent staining or TUNEL staining.

For evaluating the lung metastasis of the tumor, 4T1 (4×10^5^) were subcutaneously injected into the right flank of mice. Ten days after inoculation, mice were subjected to the same photothermal treatment as previously described for a total of three. Thirty-five days after the first treatment, mice were sacrificed and the metastatic nodules on the lungs were counted via Bouin's solution staining using double blind method 32.

### Flow cytometry analysis

Detection of MHC-I in 4T1 cells. Cells (50,000/ well) were seeded into 24 well plate and were incubated with PF, PFR, PFI or PFIR (40 μL/ well) for 8 h. Then, the cells were harvested and stained by APC anti-mouse H-2 (E-AB-F1216E, Elabscience) without permeabilization of cytomembrane. Finally, the mean fluorescence intensity (MFI) of MHC-I were detected by Flow Cytometer (FACS Verse, BD).

Assessment of DCs maturation. Tumor bearing mice were injected with hydrogels into tumor and subjected to 808 nm laser irradiation at 1.2 W/cm^2^ for 5 min. The treatment was performed every other day for 3 times. Three days after the last treatment, the draining LNs were harvested and prepared as single-cell suspensions, which were stained with CD45-PE-cy7 (103114, BioLegend), anti-mouse CD11c-APC-cy7 (117323, BioLegend), anti-mouse CD86-APC (105011, BioLegend) and anti-mouse CD80-FITC (E-AB-F0992C, Elabscience). Finally, the DCs maturation were detected by counting the percentage of CD11c^+^CD80^+^ and CD11c^+^CD86^+^ using a Flow Cytometer (FACS Verse, BD).

Assessment of CD8^+^ T cells infiltration. The tumor bearing mice underwent photothermal treatment according to the procedure of “Antitumor assessment” section. At the end point, tumors were harvested and prepared as single-cell suspensions using GentleMACS Dissociator (130-093-235, MILTENYI). Then cells were stained with anti-mouse CD45-PE-cy7 (103114, BioLegend), anti-mouse CD3-APC-cy7 (100221, BioLegend) and anti-mouse CD8a-APC (100711, BioLegend), and the percentage of CD45^+^CD3^+^CD8^+^ T cells were counted by Flow Cytometer (FACS Verse, BD).

### Statistical analysis

Data were presented as the Mean ± SD. Comparisons between the two data sets were analyzed using a Two-Tailed Student's t-test. Comparisons between three or more groups of data were analyzed using One-Way or Two-Way repeated measures analysis of variance with Tukey's post hoc test. Differences with P < 0.05, P < 0.01, and P < 0.001, were considered statistically significant and were labeled with *, **, and ***, respectively.

## Results and Discussion

### PTT evokes severe pain

**Figure 2:**
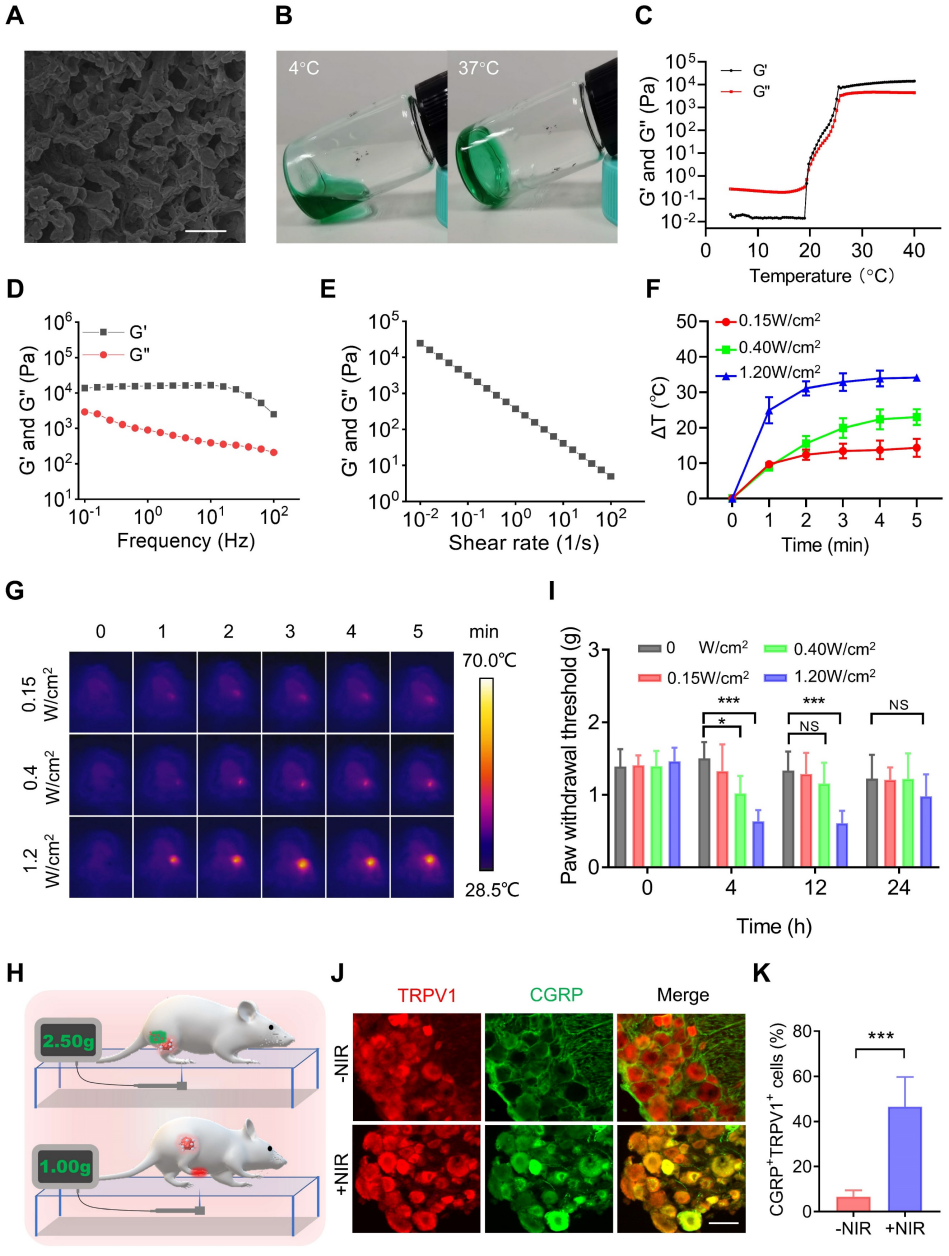
PTT using PFI evokes severe pain. (A) SEM image of PFI. Scale bar is 10 μm. (B) PFI at 4 ℃ and 37 ℃. (C) Temperature-dependent rheology of PFI aqueous dispersion. (D) Frequency-dependent rheology of PFI hydrogel at 37 °C. (E) The shear-thinning behavior of PFI hydrogel indicated by steady-shear rheology. (F) Statistical results of the degree of tumor temperature change (ΔT) (n=3). (G) IR thermal image of mice under 808 nm laser irradiation at different power densities. (H) Schematic of mechanical withdrawal threshold measurement. The force of the mouse to withdraw mechanical stimulation was recorded using the electronic Von Frey. (I) Mechanical withdrawal threshold was measured at 0, 4, 12, and 24 h after irradiation under different laser power densities (n=5). (J) Immunofluorescent staining of TRPV1 and CGRP in the dorsal root ganglion (DRG). DRGs were obtained at 6 h after PTT (1.2 W/cm^2^, 5 min). Scale bar is 50 μm. (K) Statistical results of the percentage of TRPV1^+^CGRP^+^ neurons (n=3). Data represent mean ± SD. *P < 0.05, **P < 0.01, and ***P < 0.001. 'NS' to indicate non-significance.

PTT is one of the most widespread and effective ablative treatments for tumors in preclinical studies. However, PTT is always accompanied by elevated temperatures 2 and local inflammatory responses 3, which are major causes of pain 4. Therefore, we tested the pain induced by PTT. ICG, an FDA-approved near-infrared (NIR) dye, was mixed with the PF127 hydrogel (PF127 25%, w/v) for PTT. The SEM results showed that ICG-loaded PF127 hydrogel (PFI) had a porous structure (Figure 2A). In addition, as shown in Figure 2B, the PFI solution is a free-flowing liquid at 4 °C and transforms into a hydrogel at 37 °C. This characteristic is beneficial for PFI implantation *in vivo*. Subsequently, rheological studies were performed on PFI. Storage modulus (G′) and loss modulus (G″) respectively reflected the change of viscosity and elasticity, and both of them increased with temperature (Figure 2C). Below about 19.38 ℃, G′′ was larger than G′, indicating the liquid-state behavior in these cases. By contrast, G′ > G′′ was detected upon about 19.38 ℃, which is a typical characteristic of the solid-like behavior (Figure 2C). Notably, G′ > G″ at 37 °C, suggesting a gel formation (Figure 2C). Moreover, frequency sweep at 37 °C confirms higher G′ compared to G″ for hydrogels, which showed that the formulation exhibited viscoelastic behavior (Figure 2D). Viscosity measurements were also performed, and the results showed that hydrogels exhibited a typical shear-thinning behavior (non-Newtonian) and the viscosity decreased as a function of shear rate (Figure 2E).

Furthermore, the photothermal effect of PFI were studied. The photothermal conversion efficiency of PFI was determined to be 39.08% (Figure S1). Moreover, the degree of temperature change in the mouse tumors were detected during PTT. As shown in Figure 2F and G, the tumor temperature increased by approximately 10 °C, 20 °C, and 30 °C within 5 min under the irradiation power densities of 0.15, 0.4, and 1.2 W/cm^2^, respectively. Notably, the state and thermo-responsive behavior of hydrogel were not affected by laser irradiation (Figure S2). Subsequently, the nociception in the mice right hind paw was examined by recording a mechanical stimulus-induced withdrawal response with the electronic Von Frey, a commonly used method for pain assessment 9 (Figure 2H). As shown in Figure 2I, a 10 °C increase in temperature exerted no obvious effect on the paw withdrawal threshold (PWT), but 20 °C and 30 °C increases in temperature significantly decreased the PWT for more than 4 and 12 h, respectively. The right hind paws of the mice were severely curled and were unable to touch the ground at 12 h after PTT (1.2 W/cm^2^, 5 min) (Figure S3), indicating that the severe pain lasted for more than 12 hours. Finally, the mechanisms underlying PTT-induced pain were explored. As mentioned previously, PTT can elevate temperature and induce an inflammatory response in tumors. TRPV1 is a temperature sensor and an integrator of inflammatory signals in tissues. At temperatures greater than 43 °C, TRPV1 in the primary sensory neurons is activated and induces a temperature-dependent release of neuropeptide CGRP 33. Inflammatory factors such as IL-6 can also activate TRPV1 and promote the release of CGRP, which further promotes neurogenic inflammation 34. Therefore, we tested the activation of TRPV1 by calculating the percentage of TRPV1^+^CGRP^+^ neurons in the dorsal root ganglion (DRG), where the cell body of the primary sensory neuron is located. As shown in Figure 2J-K, the percentage of TRPV1^+^CGPR^+^ cells in the DRG was significantly higher in the mice with PTT than in those without. This result suggests that TRPV1 activation is involved in PTT-evoked severe pain.

### PTT using ropivacaine-loaded hydrogel relieves therapy-induced pain

**Figure 3:**
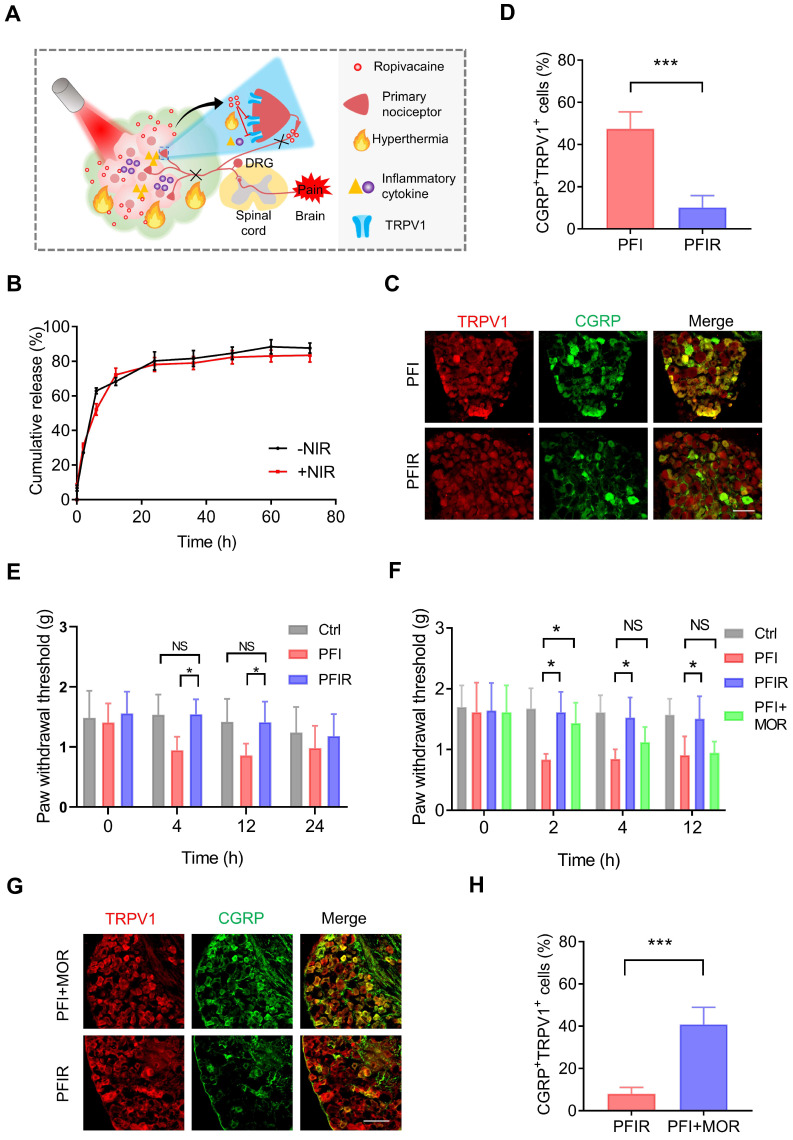
PTT with PFIR relieves therapy-induced pain. (A) Schematic of PTT with PFIR relief therapy-induced pain. Ropivacaine inhibits TRPV1 and blocks pain signal conduction. (B) Cumulative release profile of ropivacaine from PFIR with or without laser irradiation at 1.2 W/cm^2^ for 5 min. The assay was performed in triplicate. (C) Immunofluorescent staining of TRPV1 and CGRP in the dorsal root ganglion (DRG). DRGs were obtained at 6 h after PTT (1.2 W/cm^2^, 5 min). Scale bar is 100 μm. (D) Statistical results of the percentage of TRPV1^+^CGRP^+^ neurons (n=3). (E) Mechanical withdrawal threshold was measured at 0, 4, 12, and 24 h after PTT with different hydrogel (1.2 W/cm^2^, 5 min), n=6. (F) Mechanical withdrawal threshold was measured at 0, 2, 4, and 12 h after PTT with PFIR, PFI, or PFI plus additional morphine analgesia (1.2 W/cm^2^, 5 min; morphine: 5mg/ kg, SC), n=5. (G) Immunofluorescent staining of TRPV1 and CGRP in the DRG. DRGs were obtained at 6 h after PTT (1.2 W/cm^2^, 5 min). Scale bar is 100 μm. (H) Statistical results of the percentage of TRPV1^+^CGRP^+^ neurons (n=3). Data represent mean ± SD. *P < 0.05 and ***P < 0.001. 'NS' to indicate non-significance. PFI: ICG-loaded PF127 hydrogel, PFIR: ropivacaine and ICG co-loaded PF127 hydrogel, MOR: morphine.

LAs can block the pain signal conduction by inhibiting sodium channels. In addition, LAs can also inhibit TRPV1 activity directly or indirectly 35, 36. Therefore, we hypothesized that introducing LAs into PTT inhibits TRPV1 activation and prevents therapy-evoked pain (Figure 3A). However, the short duration and high toxicity at high concentrations severely limit the application of LAs. PF127 hydrogel can slow down the release of incorporated drugs 37, offering a possibility for LAs to prolong analgesia and improve safety. Indeed, we found that doping ropivacaine into PF127 hydrogel slowed down its release (Figure S4A) and reduced the incidence of convulsions induced by it (Figure S4B).

Next, ropivacaine and ICG co-loaded PF127 hydrogel (PFIR) was prepared. An abundant porous structure of the PFIR was observed using SEM (Figure S5). And PFIR exhibited the similar rheological properties to PFI (Figure S6). In addition, the release curve showed that approximately 80% of the ropivacaine was eventually released, and the plateau of the release curve was reached at about 24 h. Moreover, the release behavior of ropivacaine was not significantly different with or without laser irradiation for 5 min (Figure 3B). Besides, most of hydrogel was degraded within 48 h (Figure S7), which was consistent with previous studies 38, 39. And PFIR was found to be retained at the injection site for about 48 hours (Figure S8).

Furthermore, the prolonged relief of PTT-induced pain by PFIR were examined. As shown in Figure 3C and D, the percentage of TRPV1^+^CGRP^+^ neurons were significantly decreased in the mice underwent PTT with PFIR compared to those with PFI, indicating a less activation of TRPV1 in the PFIR-mediated PTT. Subsequently, we found that the PWT of mice underwent PTT with PFIR was significantly higher than that of mice underwent PTT with PFI for more than 12 h (Figure 3E). Notably, the PWT of PFIR-treated mice was almost comparable to that of control mice (Figure 3E). The improved posture of the mice right hind paw in the PFIR group at 12 h further supported above PWT results (Figure S9). In addition, we compared the analgesic effects of traditional opioid morphine with PIFR. As shown in Figure 3F, additional morphine analgesia remarkably increased the PWT of mice underwent PFI-mediated PTT at 2 h, and this effect disappeared after 4 h. However, the PWT was significantly increased by more than 12 h in mice receiving PFIR-mediated PTT compared to mice receiving PFI-mediated PTT (Figure 3F). Additionally, the percentage of TRPV1^+^CGRP^+^ neurons in the mice receiving PFIR-mediated PTT were significantly lower than those receiving PFI-mediated PPT plus additional morphine analgesia (Figure 3G-H), confirming the long-lasting analgesia of PFIR. These findings suggest that PTT using PFIR continuously inhibits severe pain evoked by PTT.

In clinical settings, only a subset of patients is suitable for ablation, while most patients who do not undergo ablation will also experience pain from cancer. Interestingly, in a mouse model of cancer pain, we found a significant increase in PWT for more than 12 h in mice treated with ropivacaine-loaded PF127 hydrogel (PFR) compared to those untreated (Figure S10A). In addition, tumor-bearing mice curl up their paws due to pain evoked by tumor compression, while mice treated with PFR exhibited normal paw posture for more than 12 h (Figure S10B). These results revealed that ropivacaine-loaded PF127 hydrogel also provides a sustained relief of spontaneous cancer pain in the mice.

### Ropivacaine-loaded hydrogels increase MHC-I *in vitro*

**Figure 4:**
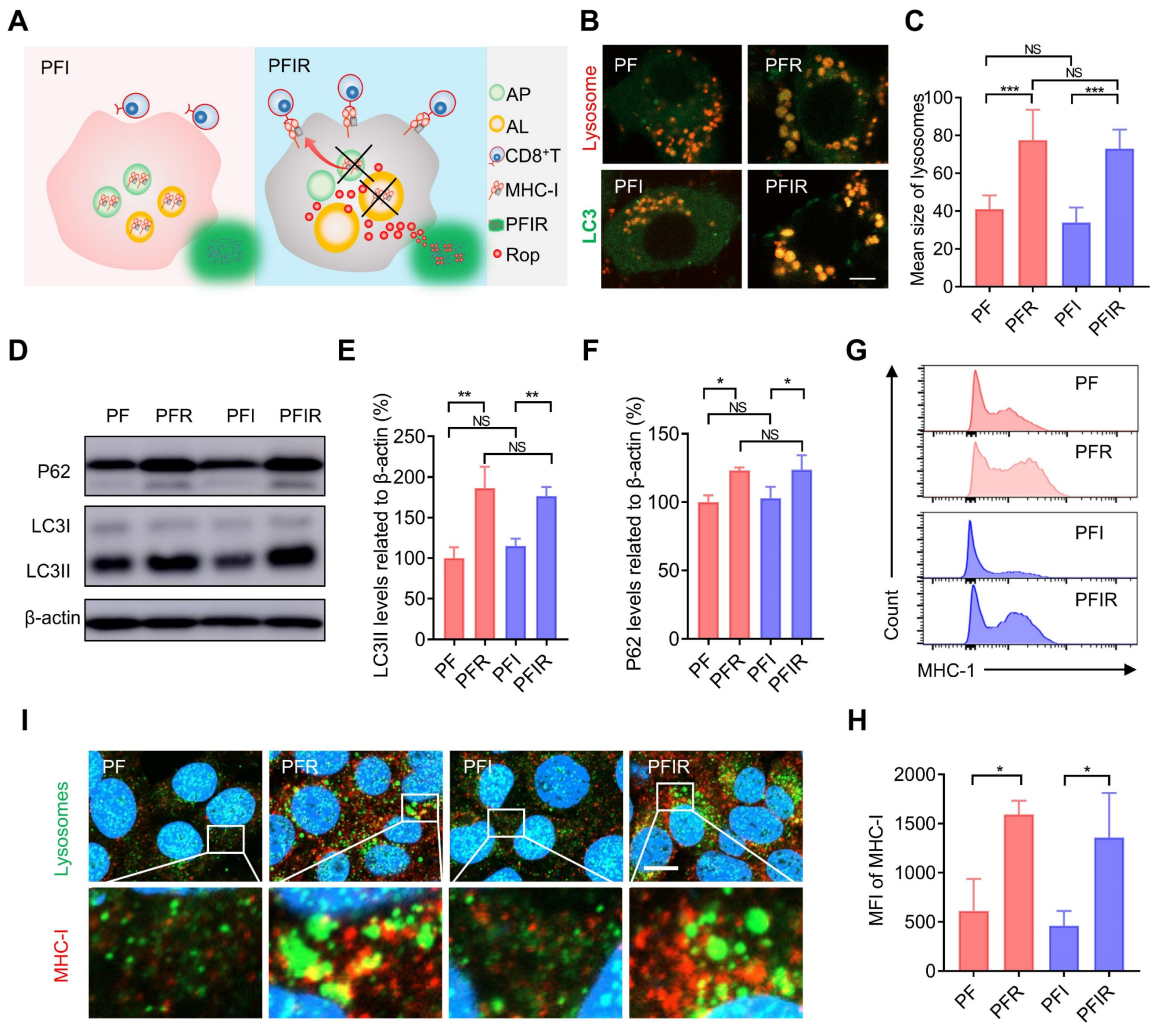
Ropivacaine-doped hydrogels increase MHC-I in tumor cells. (A) Schematic of MHC-I upregulation by ropivacaine-loaded hydrogels. AP: autophagosome, AL: autolysosome, Rop: ropivacaine. (B) Fluorescent image of LC3 and lysosomes. Ad-GFP-LC3B-infected 4T1 cells were treated with PF, PFR, PFI, or PFIR for 8 h and then stained with Lyso-Tracker Red. Scale bar is 5 μm. (C) Statistical results of the size of lysosomes from (B). (D-F) Western blot and statistical results of LC3II and P62. Cells were subjected to different treatments for 8 h. (G) Flow cytometry results of the mean fluorescence intensity (MFI) of MHC-I in 4T1 cell. (H) Statistical results from (G). (I) Immunofluorescent staining of MHC-I (red), LAMP2 (green), and DAPI (blue). Scale bar is 5 μm. All the experiments were repeated three times. Data represents mean ± SD. *P < 0.05, **P < 0.01 and ***P < 0.001. 'NS' to indicate non-significance. PF: PF127 hydrogel, PFR: ropivacaine-loaded PF127 hydrogel, PFI: ICG-loaded PF127 hydrogel, PFIR: ropivacaine and ICG co-loaded PF127 hydrogel.

Aside from analgesic applications, the antitumor efficacy of LAs remains to be explored. For instance, whether LAs can mobilize the immune system to facilitate tumor therapy is unclear. Impaired antigen presentation caused by the loss of MHC-I is a common mechanism of immune evasion by tumor cells 23. Keisuke et al. found that MHC-I is selectively targeted for lysosomal degradation via an autophagy-dependent mechanism. Impairing autophagy restores MHC-I and leads to improved antigen presentation, enhanced antitumor T cell responses, and reduced tumor growth in mice 23. Our previous study showed that ropivacaine impairs autophagic lysosomal degradation in tumor cells 14. Therefore, we hypothesized that ropivacaine upregulates MHC-I levels in tumor cells (Figure 4A). Enlarged autophagosomes usually indicate impaired autophagic lysosomal degradation 40. Consistent with the results for ropivacaine 14, PFR and PFIR induced many enlarged autolysosomes labeled by co-staining with LC3 and lysosomes (Figure 4B). Statistical results also showed a significant increase in the size of lysosomes in the cells treated with PFR or PFIR (Figure 4C). LC3II is a marker of autophagosomes 41. While P62 is a protein substrate that is selectively degraded by autophagy 42, and its level indicates the capacity of autophagic lysosomes degradation. Consistent with the results for ropivacaine treatment 14, PFR and PFIR treatment significantly increased the levels of LC3II and P62 compared with their respective controls (Figure 4D-F), further confirming the impairment of autophagy. Notably, compared with PF treatment, PFI treatment did not increase the size of lysosomes and P62 levels, ruling out the possibility of ICG disrupting autophagy. Subsequently, MHC-I levels were measured in cells. We found that free ropivacaine increased the mean fluorescence intensity (MFI) of MHC-I in cells in a dose- and time-dependent manner (Figure S11). Furthermore, as shown in Figure 4G-H, the MFI of MHC-I in cells was significantly increased in the PFR- and PFIR-treated cells compared with their respective controls. In addition, we observed via confocal microscopy the enlarged lysosomes and stronger fluorescence signals of MHC-I in the PFR- and PFIR-treated cells compared with their respective controls (Figure 4I). These results demonstrate that ropivacaine-loaded hydrogels upregulate the levels of MHC-I in cells.

### Photothermal therapy using ropivacaine and imiquimod co-loaded hydrogels

**Figure 5:**
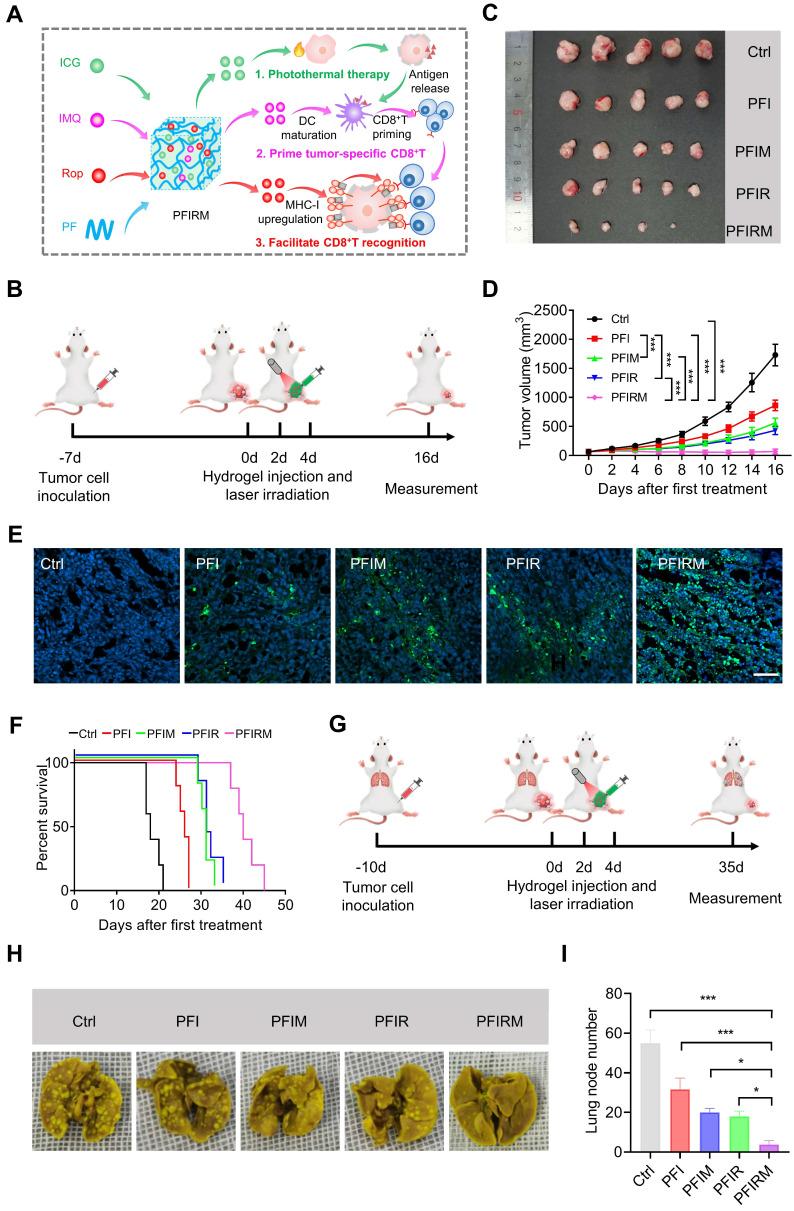
Antitumor effect of PTT with PFIRM. (A) Schematic design of hydrogel. IMQ: imiquimod, Rop: ropivacaine, PF: PF127. (B) Schematic showing the experimental operation procedure for testing the inhibition of tumor growth. (C) Tumors removed from mice after indicated treatment. (D) Average tumor growth curves (n=5). (E)TUNEL staining of tumor sections. Nuclei were stained with DAPI. Scale bar is 50 μm. (F) Survival curves of tumor-bearing mice after indicated treatment (n=5). (G) Schematic showing the experimental operation procedure for testing the inhibition of tumor metastasis to the lung. (H) Representative images of lung of mice after indicated treatment. (I) Statistical results (n=3) from (H). Data represent mean ± SD. *P < 0.05, **P < 0.01 and ***P < 0.001. 'NS' to indicate non-significance. PFI: ICG-loaded PF127 hydrogel, PFIR: ropivacaine-loaded PFI hydrogel, PFIM: imiquimod-loaded PFI hydrogel, PFIRM: imiquimod and ropivacaine co-loaded PFI hydrogel.

Ropivacaine upregulates MHC-I in tumor cells, suggesting its immunotherapy potential. Therefore, we rationally synthesize a PF127 hydrogel co-doped with ropivacaine, TLR7 agonist imiquimod and ICG (PFIRM) for potentiated tumor photothermal therapy. As previously reported, imiquimod combined with tumor antigens can promote DCs maturation, subsequently priming and proliferating tumor specific CD8^+^ T cells 2, 25, 26. After this, CD8^+^ T cells specifically recognize tumor cells through MHC-I on them and kill them 22, 23. Hence, we hypothesized that PFIRM can potentiate photothermal therapy through synergistically priming tumor-specific CD8^+^ T cells and facilitating their recognition of tumor cells (Figure 5A). To test the hypothesis, we first prepared the PFIRM which showed similar rheological properties to PFI (Figure S12). Besides, the plateau of the release curve of imiquimod and ICG were reached at about 24 h, and the release behavior of drugs were not significantly different with or without laser irradiation for 5 min (Figure S13). Then, the killing effect of PFIRM-mediated photothermal treatment on tumor cells was tested *in vitro*. As shown in Figure S14A-B, most of cells were killed and the cell viability were also significantly decreased after the photothermal treatment using PFIRM. In addition, the concentration of released ATP and HMGB-1 from cells and the levels of calreticulin (CRT) on cell surface were significantly increased after photothermal treatment using PFIRM (Figure S14C-E), which demonstrated the induction of immunogenic cell death (ICD). Notably, the effects of cell killing and ICD induction by PFIRM were similar to those of other hydrogels.

Furthermore, the efficacy of PTT using PFIRM was assessed in subcutaneous tumor-bearing mice (Figure S15). Mice were randomly divided into five groups and subjected to PTT for three times (Figure 5B and Figure S16). As shown in Figure 5C, the tumors of PFIRM-treated mice were the smallest among all groups. In addition, PTT using PFIR or imiquimod-loaded PFI (PFIM) showed mild antitumor effects, whereas PTT using PFIRM exerted strong antitumor effects (Figure 5D). Specifically, the tumor growth in PFIRM-treated mice was significantly slower than that in PFI-, PFIR-, or PFIM-treated mice, indicating that different components in the hydrogel had a synergistic effect against tumors (Figure 5D). Furthermore, TUNEL assay showed the most obvious tumor cell apoptosis following PTT with PFIRM (Figure 5E). Notably, there were no significant differences in body weight, physiological structure and function of the major organs of the mice between the different treatment groups, suggesting that PTT using hydrogels had a good biosafety profile (Figure S17). Moreover, the survival curves of the tumor-bearing mice were plotted. As shown in Figure 5F, PTT using PFIRM significant extended the median survival time of mice to 40 days which was longer than that of any other group of mice. Notably, the median survival time of cisplatin-treated mice was 24 days which was much shorter than that of PFIRM-treated mice (Figure S18). Besides, we examined the metastasis of tumors to the lungs in mice after different treatment (Figure 5G). The results showed that PFIRM-treated mice had significantly fewer lung nodules than the other groups (Figure 5H-I). These results indicate that PFIRM-mediated PTT has strong antitumor effects.

In addition, we tested the antitumor effect of PTT using PFIRM in cancer pain mice that were inoculated tumor cells in deeper muscular tissue (Figure S15). Tumor-bearing mice were grouped and treated as above (Figure 5B). Mice underwent PTT using PFIRM showed smallest tumor size among all groups (Figure S19A). Moreover, the tumor growth in PFIRM-treated mice remarkably slower than that in PFI-, PFIR- and PFIM-treated mice (Figure S19B-C). PTT with PFIRM also induced the most obvious tumor cells apoptosis among all the treatment (Figure S19D).

### Mechanism validation of photothermal therapy

**Figure 6:**
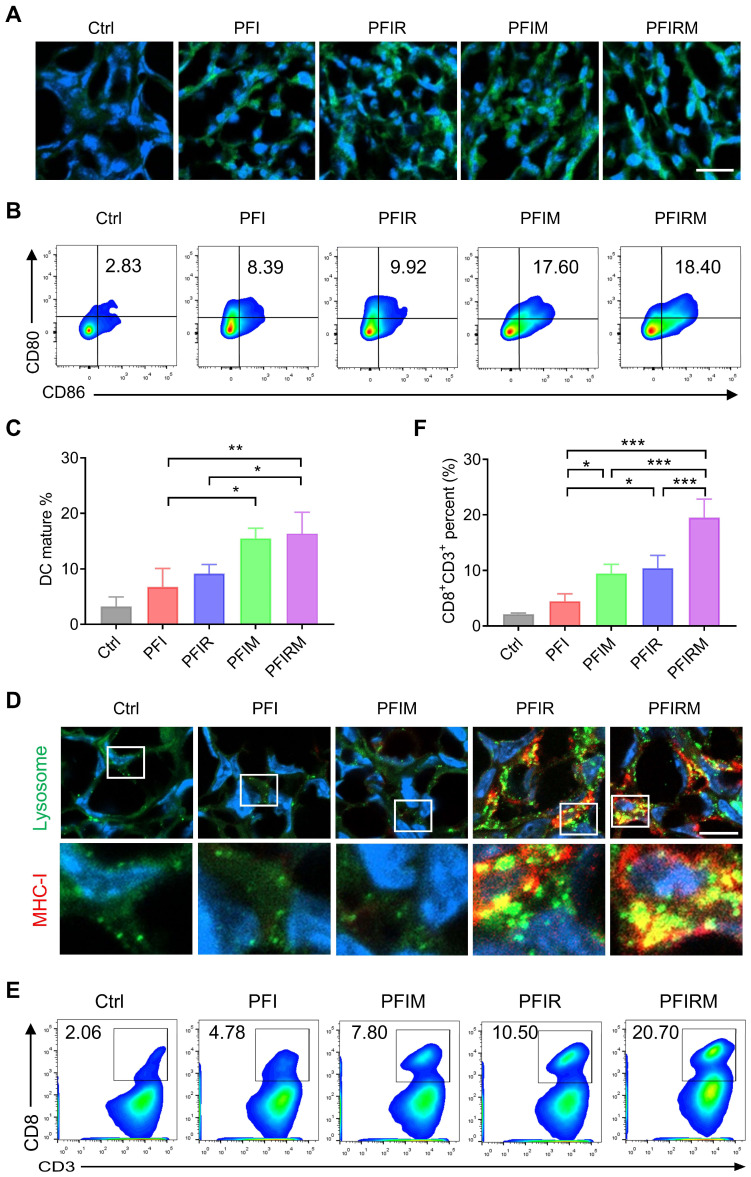
Mechanism validation of photothermal therapy with PFIRM. (A) Immunofluorescent staining of CRT in tumor. Scale is 20 μm. (B-C) Flow cytometry analysis of the percentage of CD80^+^CD86^+^ DCs in draining lymph nodes (n=3). (D) Immunofluorescent staining of MHC-I and LAMP2 in tumor. Scale bar is 10 μm. (E-F) Flow cytometry analysis of the percent of CD8^+^CD3^+^ T cells in tumors (n=3). Data represent mean ± SD. *P < 0.05, **P < 0.01 and ***P < 0.001. 'NS' to indicate non-significance. PFI: ICG-loaded PF127 hydrogel, PFIR: ropivacaine-loaded PFI hydrogel, PFIM: imiquimod-loaded PFI hydrogel, PFIRM: imiquimod and ropivacaine co-loaded PFI hydrogel.

As imiquimod can prime and proliferate tumor specific CD8^+^ T cells by promoting DCs maturation 2, 25, 26, the DCs maturation and percentage of CD8^+^ T cells population were examined. But before that, ICD induction can result in the release of “eat me” clues and tumor-associated antigens, assisting imiquimod to promote DCs maturation and priming tumor specific CD8^+^ T cells 25. Therefore, we detected the ICD induction by PTT and found that the CRT expression in tumor tissues was obviously increased in mice receiving PTT with hydrogels (Figure 6A). Then the percentage of CD80^+^CD86^+^ DCs were examined. The results showed that the percentage of CD80^+^CD86^+^ DCs in lymph nodes, spleen and tumors are significantly higher in PFIM- and PFIRM-treated mice compared with PFI-treated mice (Figure 6B-C, Figure S20). What's more, the infiltration of CD8^+^ T cells into tumors are also significantly increased in PFIM- and PFIRM-treated mice compared to PFI-treated mice (Figure 6 E-F). Following CD8^+^ T cell priming, specific recognition of tumor cells by CD8^+^ T cells is governed by MHC-I on tumor cells 22, 23. Hence, we next examined the MHC-I in mice tumor. We found that PTT with PFIR and PFIRM induce many enlarged lysosomes and upregulate MHC-I in the mice tumor (Figure 6D). Consequently, the infiltration of CD8^+^ T cells into tumor were significantly increased in PFIR- and PFIRM-treated mice compared to PFI-treated mice (Figure 6E-F). Notably, the mice treated with PFIRM showed the highest percentage of CD8^+^ T cells in tumor tissues (Figure 6 E-F), demonstrating the synergistic effect of different components of PFIRM on T cell immunity. In addition, the similar enhanced T cell immunity was also validated in intramuscular tumor-bearing mice after PTT using PFIRM (Figure S21). Collectively, PTT using PFIRM synergistically prime tumor specific CD8^+^ T cells and facilitate their recognition of tumor cells, maximumly promoting CD8^+^ T cells infiltration into tumor tissues.

## Conclusion

In summary, PTT using ICG-loaded PF127 hydrogel (PFI) induces severe pain in tumor-bearing mice, which depends on an increase in tumor temperature. In the meanwhile, TRPV1 in DRG is activated by PTT. However, PTT with ropivacaine-loaded PFI (PFIR) relieves severe therapy pain and inhibits TRPV1 activation. Moreover, a ropivacaine, imiquimod and ICG co-loaded PF127 hydrogel (PFIRM) synergistically prime tumor-specific CD8^+^ T cells and facilitate their recognition of tumor cells, enhancing the PTT effect on tumor. Overall, this study for the first time provides an LA-based approach for analgesic and potentiated photothermal therapy.

## Supplementary Material

Supplementary methods and figures.
