# Supplementary material for: Analgesic and potentiated photothermal therapy with ropivacaine-loaded hydrogels
Source: Theranostics. 2023 Apr 9;13(7):2226–40. doi: 10.7150/thno.81325 (PMC10157729; doi:10.7150/thno.81325)
Supplement: Supplementary file 1 — Supplementary methods and figures. [file thnov13p2226s1.pdf]

Supplementary Materials

Methods

In vitro hydrogel degradation

The in vitro degradation of hydrogels was determined by a weight remaining (%) experiment described by previous literature[1]. Briefly, samples of 500 μL of PFIR hydrogel formulations were added into glass vials and placed at 37 °C. After gelation, the original weight of each hydrogel formulation was recorded as W0. 1 mL of pre-equilibrated PBS (pH 7.4) was gently laid over the surface of the hydrogels and incubated in a metal bath at 37 °C. The weight of the remaining hydrogel samples (Wt) was recorded at regular time intervals (1, 3, 6, 9, 12, 24, 36, and 48 h) after removing the supernatant. The *in vitro* degradation assay was performed in triplicate. The weight remaining (%) was calculated as: $Weight\ remaining$ (%) = $Wt\ /\ W0$ × 100%.

Degradation of hydrogel in vivo

100 μL of PFIR hydrogel subcutaneously injected into the right flank of BALB/c mice. At indicated timepoints, the mice were sacrificed, and the residual hydrogel in the subcutaneous layer was photographed.

In vitro cytotoxicity evaluation

Cells (10, 000/ well) were seeded into 96-well plate and cultured with DMEM medium supplemented with 10% FBS. Next day, cells were treated with PBS, PFI, PFIR, PFIRM or PFIRM (10 μL / well), and then irradiated with 808 nm laser at 1.2 W/ cm^2 for 5 min. Twenty-four hours after laser irradiation, cell viability were assayed using a CCK-8 detection kit (BS350A, Biosharp,) according to the manufacturer's instructions. The absorbance at 450 nm was measured using a microplate reader (Infinite M200 Pro, 459 Tecan). For cell death detection, 24 hours after irradiation, cells were stained using a Calcein-AM/PI kit (CA1630, Solarbio) according to the manufacturer's instructions. Stained cells were observed under a fluorescence microscope (Olympus IX71, Olympus).

In vitro immunologic cell death (ICD) induction

Cells (50, 000/ well) were seeded into 24 well plate and cultured with DMEM medium supplemented with 10% FBS. Next day, cells were treated with PBS, PFI, PFIR, PFIRM

or PFIRM (40 μL / well), and then irradiated with 808 nm laser at 1.2 W/ cm^2 for 5 min. Twenty-four hours after laser irradiation, the medium were collected and centrifuged at 10,000 rpm for 5 min. The supernatant was collected and assayed using an ATP Content Assay Kit (BC0300, Solarbio) or an HMGB-1 ELISA Detection Kit (CSB-E08225m, CUSABIO) according to the manufacturer's instructions. For calreticulin (CRT) detection, cells (50, 000/ well) were seeded into 24 well plate with glass bottom and cultured with DMEM medium supplemented with 10% FBS. Next day, cells were treated with different hydrogels and irradiated with 808 nm laser at 1.2 W/ cm^2 for 5 min. Twenty-four hours after irradiation, the cells were rinsed twice with PBS and fixed in 4 % paraformaldehyde (PFA) for 10 min and then rinsed twice with PBS, incubated with Alexa Fluor® 488 Anti-Calreticulin antibody (1:500, ab196158, Abcam) overnight at 4 °C. Then, the cells were rinsed 3 times with PBS. The cells after immunofluorescent staining were observed at a confocal fluorescence microscope (LSM800, Zeiss).

Detection of the relief of cancer pain by PFR

Mice were anesthetized with isoflurane. One million 4T1 cells in 100 μL of sterile PBS were injected into the muscular tissue in the immediate vicinity of the nerve near the trochanter[2]. Ten days after inoculation, the paw was observed to curl up, indicating the successful establishment of a mouse model of spontaneous cancer pain. To test the analgesic effect, 100 μL 10% (w/v) free ropivacaine or PFR containing 10% ropivacaine (w/ v) was injected into the tumor near the sciatic nerve[3]. Then the posture of mouse paw was observed and mechanical pain was tested at indicated timepoint after administration.

Results

Figure S1

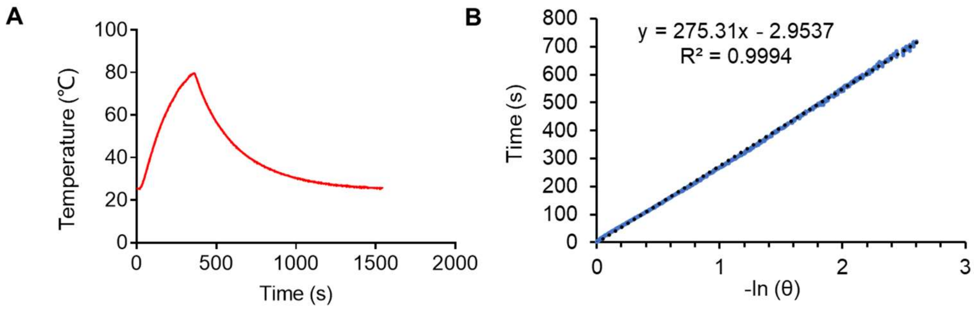

Figure S1. (A) Photothermal effect of PFI under 808nm laser irradiation (1.2 W/cm^2). (B) The cooling time versus the negative natural logarithm of the temperature driving force obtained from the cooling stage. The time constant for the heat transfer of the system was determined to be τ (s) =275.31 (s). The photothermal conversion efficiency of PFI was determined according to pervious reports [4, 5].

Figure S2

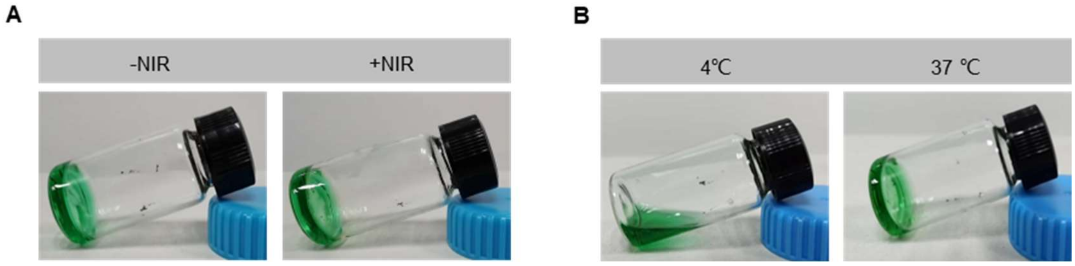

Figure S2. (A) Image of PFI at 37 ℃ with or without laser irradiation (1.2 W/cm^2, 5min). (B) The state of PFI at 4 ℃ or 37 ℃ following a laser irradiation at 1.2 W/cm^2 for 5min.

Figure S3

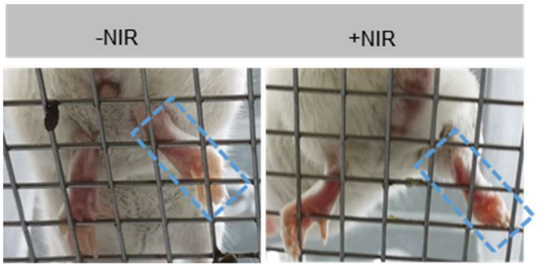

Figure S3. Paws of tumor-bearing mice at 12 h after PTT with PFI (1.2 W/cm^2, 5 min).

Figure S4

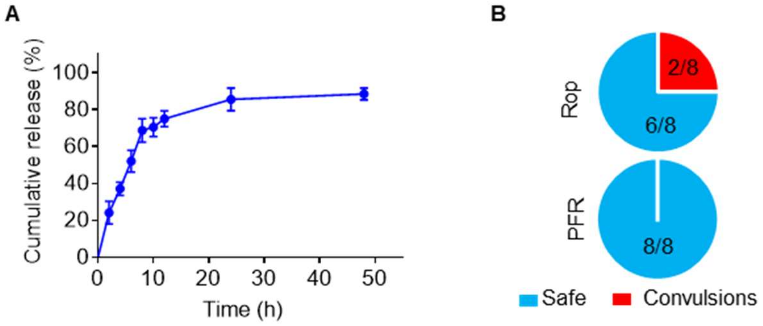

Figure S4. (A) Cumulative release profile of ropivacaine from PFR. The assay was performed in triplicate. (B) Statistical results of the occurrence of convulsions in tumor-bearing mice after PFR or free ropivacaine intratumor injection (n=8). PFR: ropivacaine-loaded PF127 hydrogel.

Figure S5

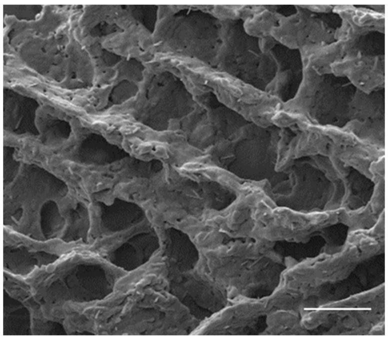

Figure S5. SEM image of PFIR. Scale bar is 10 μm.

FigureS6

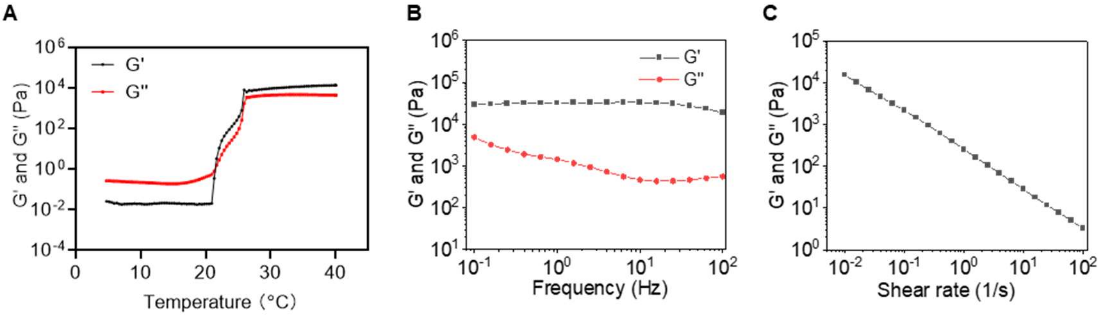

Figure S6. Rheology properties of PFIR. (A) Temperature-dependent rheology of PFIR aqueous dispersion. (B) Frequency-dependent rheology of PFIR hydrogel at 37 °C. (C) The shear-thinning behavior of PFIR hydrogel indicated by steady-shear rheology.

FigureS7

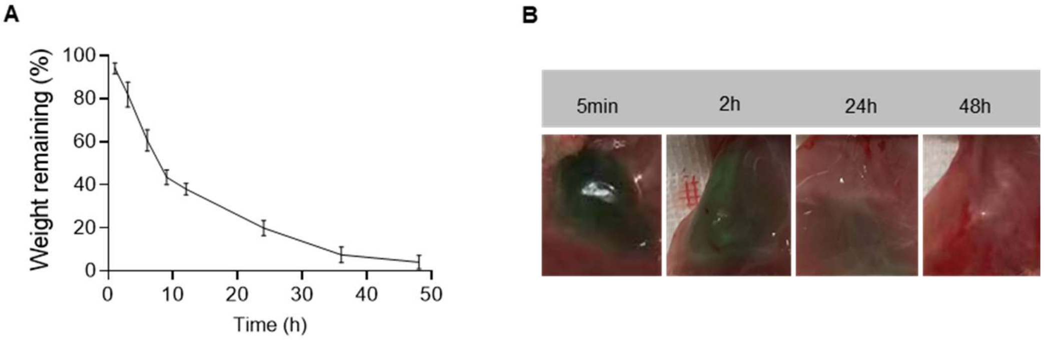

Figure S7. (A) Degradation curve of PFIR incubated at 37 °C measured by weight remaining (%). The assay was repeated three times. (B) Degradation of PFIR in vivo. The PFIR was injected into mice subcutaneously and photos around the hydrogels were taken at 5 min, 12, 24 and 48 h after injection.

Figure S8

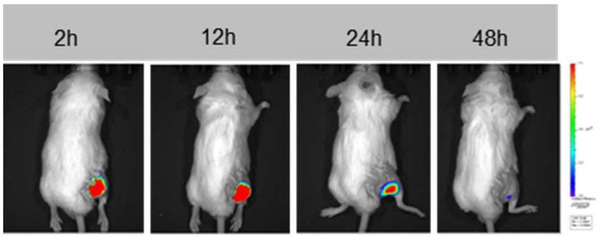

Figure S8. Fluorescence images of mice at the indicated times after subcutaneous injection of 100 μL PFIR.

Figure S9

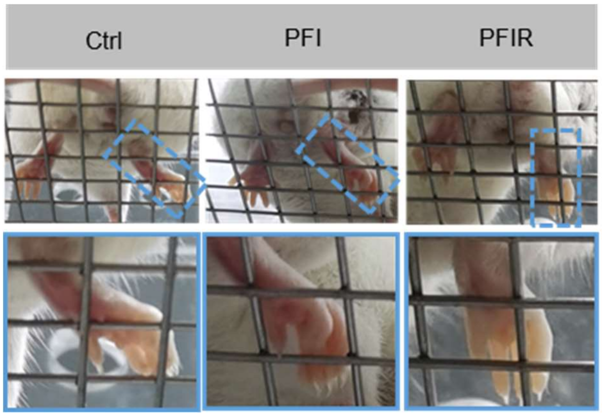

Figure S9. Paws of tumor-bearing mice at 12 h after PTT with PFI or PFIR (1.2 W/cm^2, 5 min).

Figure S10

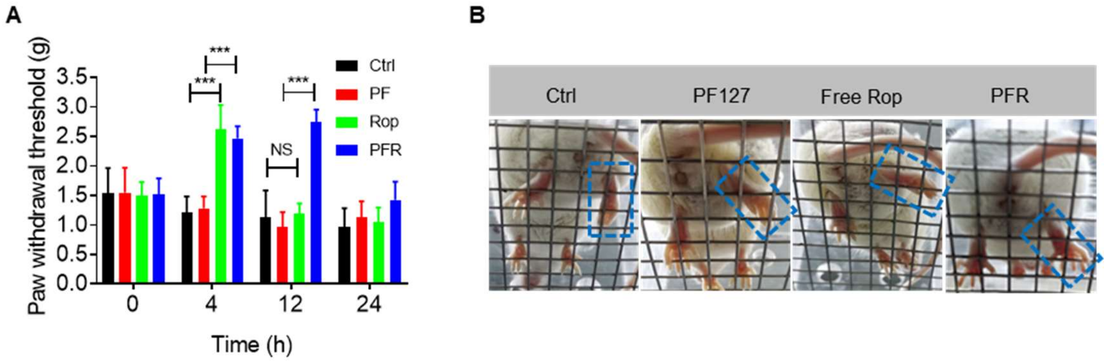

Figure S10. (A) Mechanical withdrawal threshold was measured at 0, 4, 12, and 24 h after different treatments (n=5). (B) Paws of tumor-bearing mice under different treatments for 12 h. Data represent mean ± SD. ***P < 0.001. 'NS' to indicate non-significance.

Figure S11

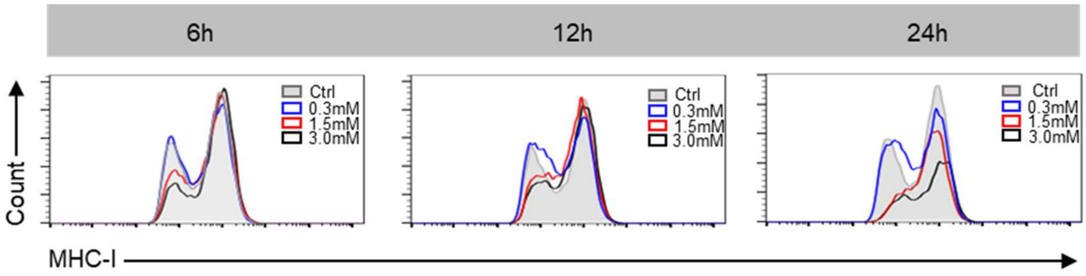

Figure S11. Flow cytometry results of the mean fluorescence intensity (MFI) of MHC-I in 4T1 cell. Cells were treated with 0.3, 1.5 or 3.0 mM of ropivacaine for 6, 12 or 24 hours respectively.

Figure S12

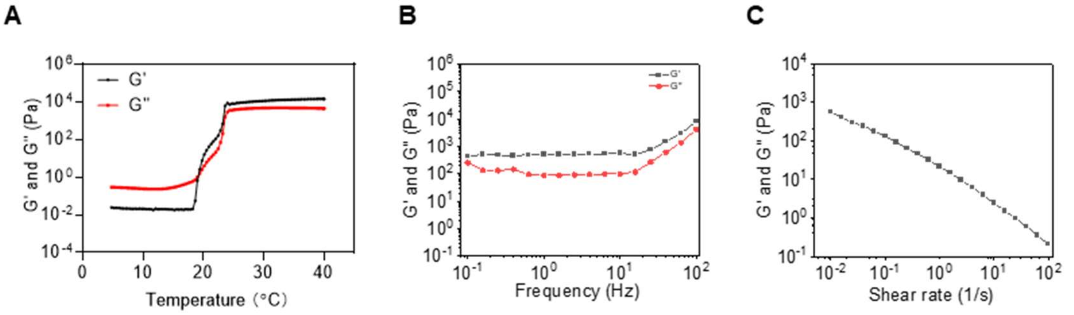

Figure S12. Rheology properties of PFIRM. (A) Temperature-dependent rheology of PFIRM aqueous dispersion. (B) Frequency-dependent rheology of PFIRM hydrogel at 37 °C. (C) The shear-thinning behavior of PFIRM hydrogel indicated by steady-shear rheology.

Figure S13

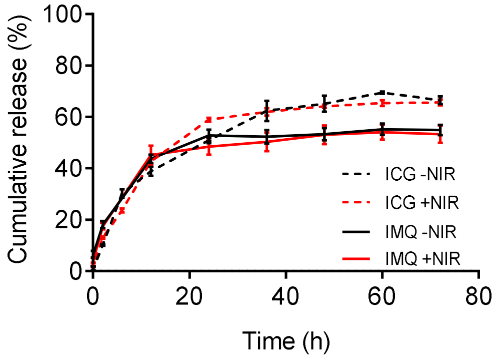

Figure S13. Cumulative release profile of ICG and imiquimod (IMQ) from PFIRM with or without laser irradiation at 1.2 W/cm^2 for 5 min. The absorbance of IMQ and ICG were measured at 320 and 800 nm, respectively, using a Micro UV-Vis Spectrophotometer (LIFEREAL, FC-1100). The assay was repeated three times.

Figure S14

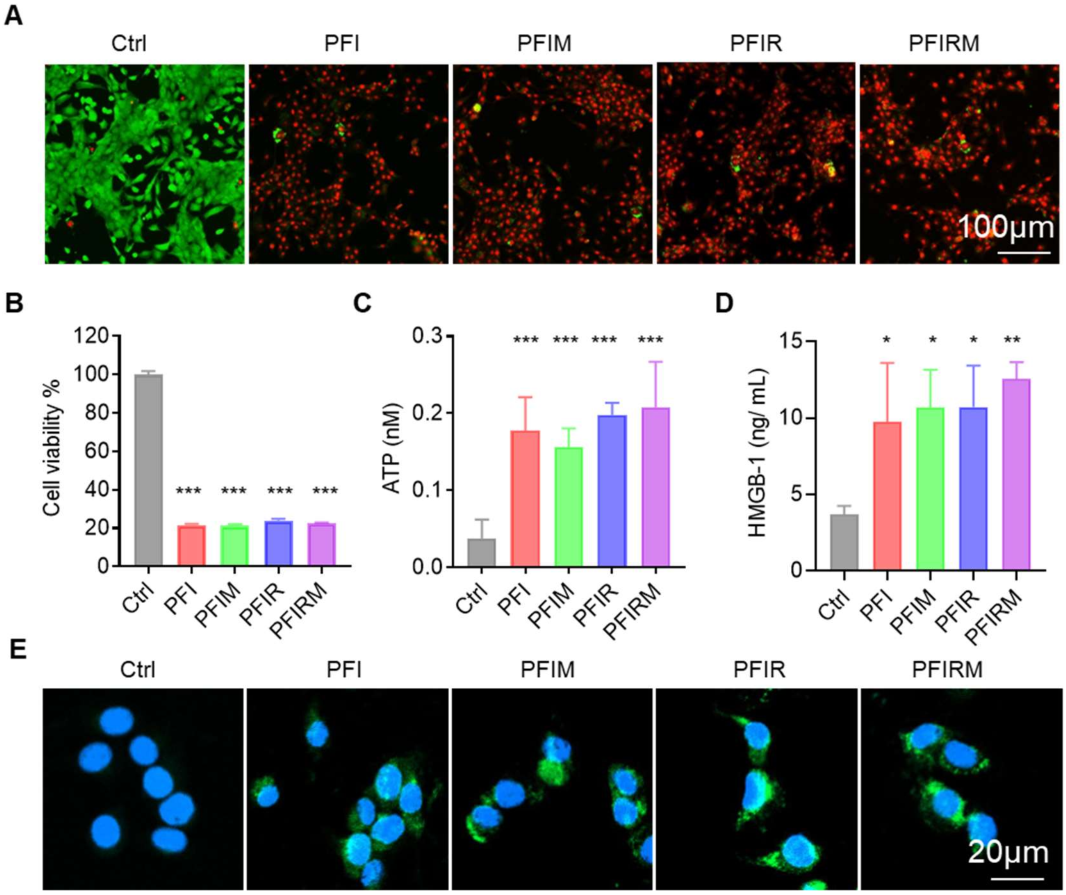

Figure S14. In vitro cytotoxicity evaluation. (A) Live/dead staining of 4T1 cells with Calcein-AM/PI. (B) Cell viability of 4T1 cells was tested using CCK-8 method. Detection of (C) ATP and (D) HMGB-1 extracellular release. (E) Fluorescent imaging of ecto-CRT expression on the surface of 4T1 cells. After incubated with 40 μL of PBS,

PFI, PFIM, PFIR or PFIRM in 500 μL DMEM culture medium, cells were subjected to laser irradiation at 1.2 W/cm^2 for 5 min. Twenty-four hours after irradiation, cells were stained, and the concentration of ATP or HMGB-1 in culture medium were quantified. The assay was repeated three times. Data represents mean ± SD. *P < 0.05, **P < 0.01 and ***P < 0.001. 'NS' to indicate non-significance.

Figure S15

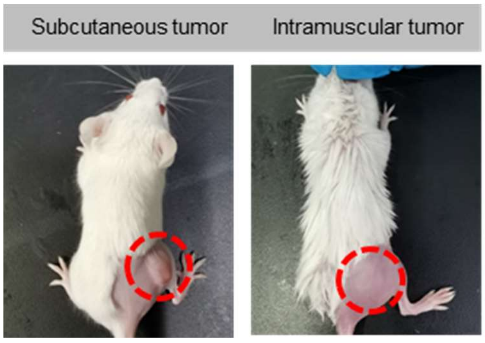

Figure S15. Photos of tumor-bearing mice. Red circle indicates the site of tumor. The construction of intramuscular tumor-bearing mice was same to that of cancer pain mice.

Figure S16

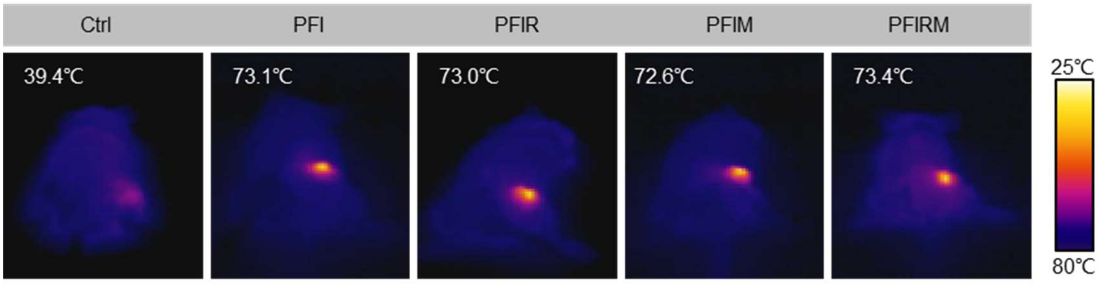

Figure S16. IR thermal image of subcutaneous tumor-bearing mice underwent indicated hydrogels treatment following 808 nm laser irradiation (1.2W/ cm^2, 5 min). The tumor temperatures were recorded using a Thermal Infrared Imager (H10, HIKVISION) with IVMS-4800 software.

Figure S17

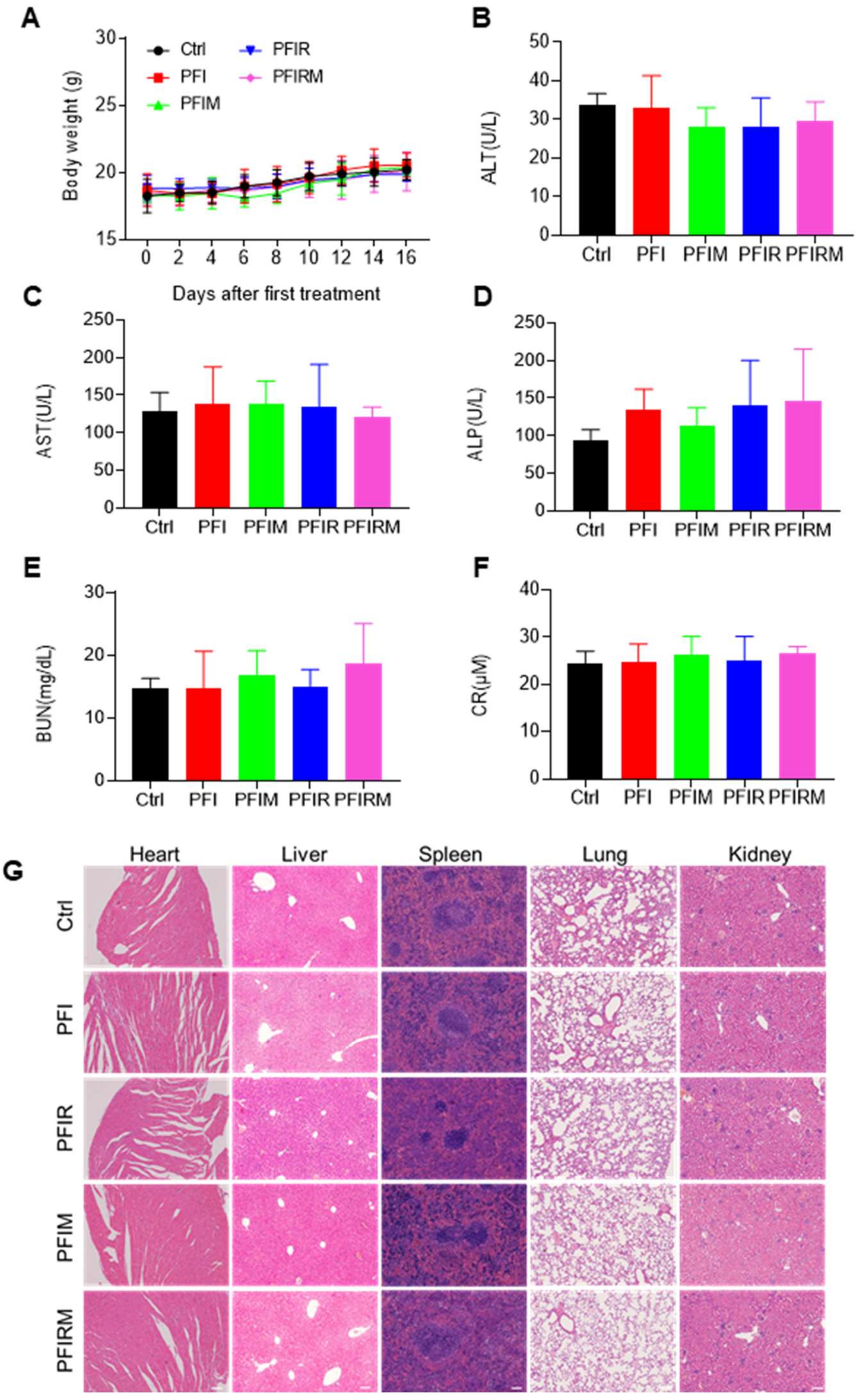

Figure S17. Biosafety assessment of PTT with hydrogels. (A) Body weight changes in mice under different treatments. (B-F) Blood aspartate aminotransferase (AST), alanine aminotransferase (ALT), alkaline phosphatase (ALP), urine nitrogen (BUN) and creatinine (CR) values. (G) HE staining of major organs. The tumor-bearing mice were randomly grouped, and were subjected to PBS, PFI, PFIR, PFIM or PFIRM treatment according to previous operation procedure (Figure 5B). Body weight changes were recorded. After therapy, mice were sacrificed. Mice tumors and serum were respectively conducted HE staining and biochemistry assessment by Servicebio (Wuhan, China). Scale bar is 1mm.

Figure S18

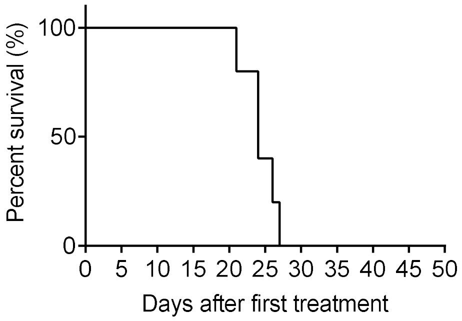

Figure S18. Survival curve of tumor-bearing mice after cisplatin treatment (n=5). Subcutaneous tumor-bearing mice (tumor size reach about 60 mm^3) received a single dose of the 7.0 mg/kg cisplatin intraperitoneally every other day for total three times.

Figure S19

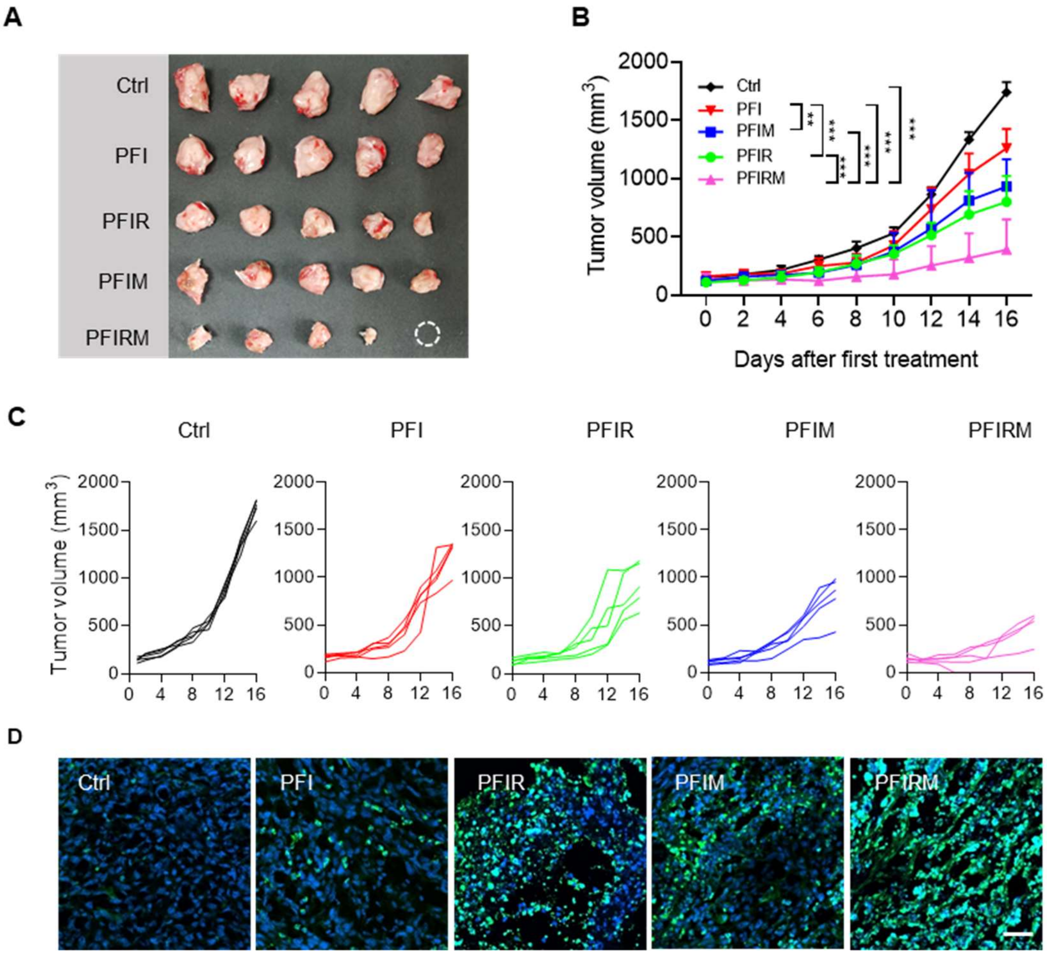

Figure S19. Antitumor effect of PTT with PFIRM in intramuscular tumor-bearing mice. (A) Exfoliated tumor. (B) Average tumor growth curves (n=5). (C) Individual tumors monitored in mice after indicated treatments. (D) TUNEL staining of tumor sections. Nuclei were stained with DAPI. Scale bar is 50 μm. Data represent mean ± SD. *P < 0.05, **P < 0.01 and ***P < 0.001.

Figure S20

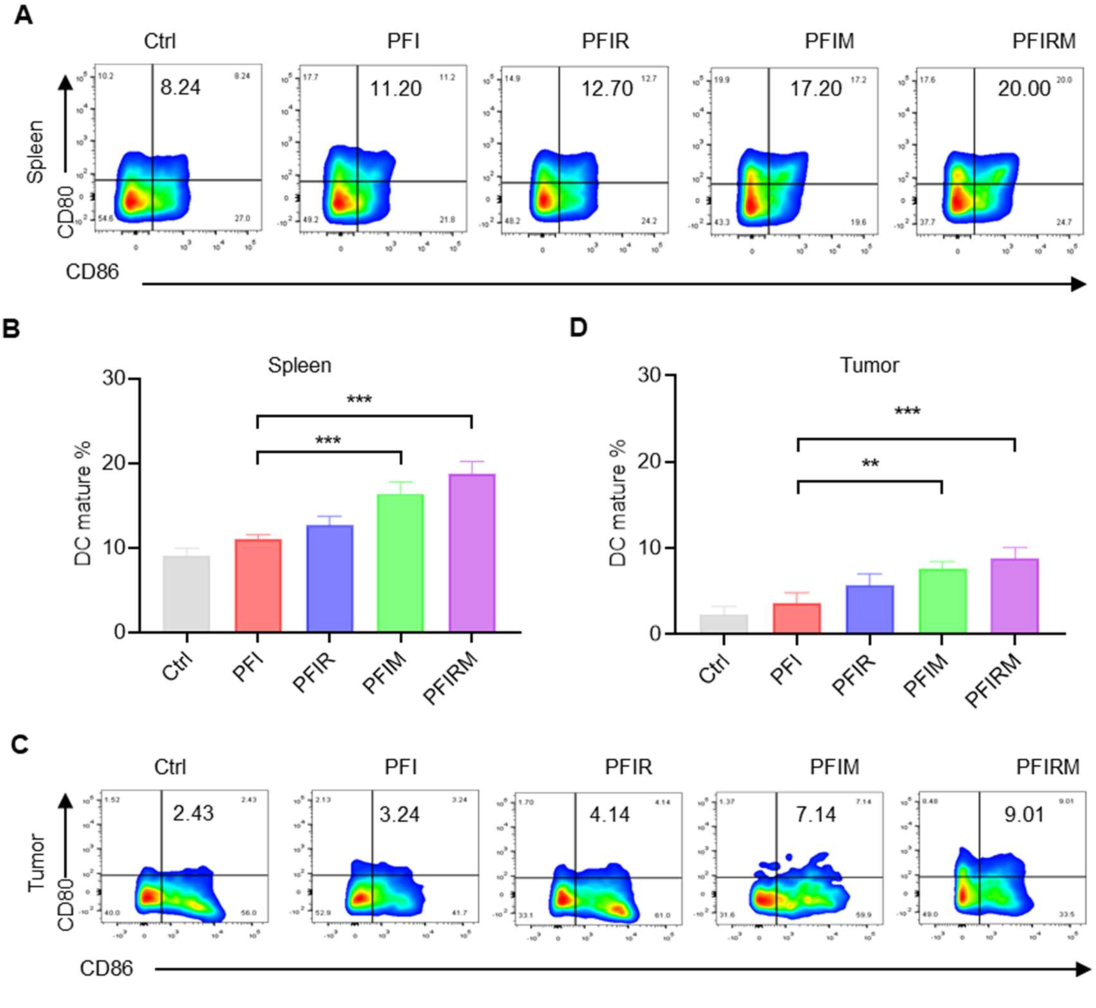

Figure S20. Flow cytometry analysis of the percentage of $CD80^{+}CD86^{+}$ DCs in (A-B) spleen or (C-D) tumors (n=3). Data represent mean ± SD. $^{**}P < 0.01$ and $^{***}P < 0.001$. The treatment and flow cytometry analysis refer to "Materials and method".

Figure S21

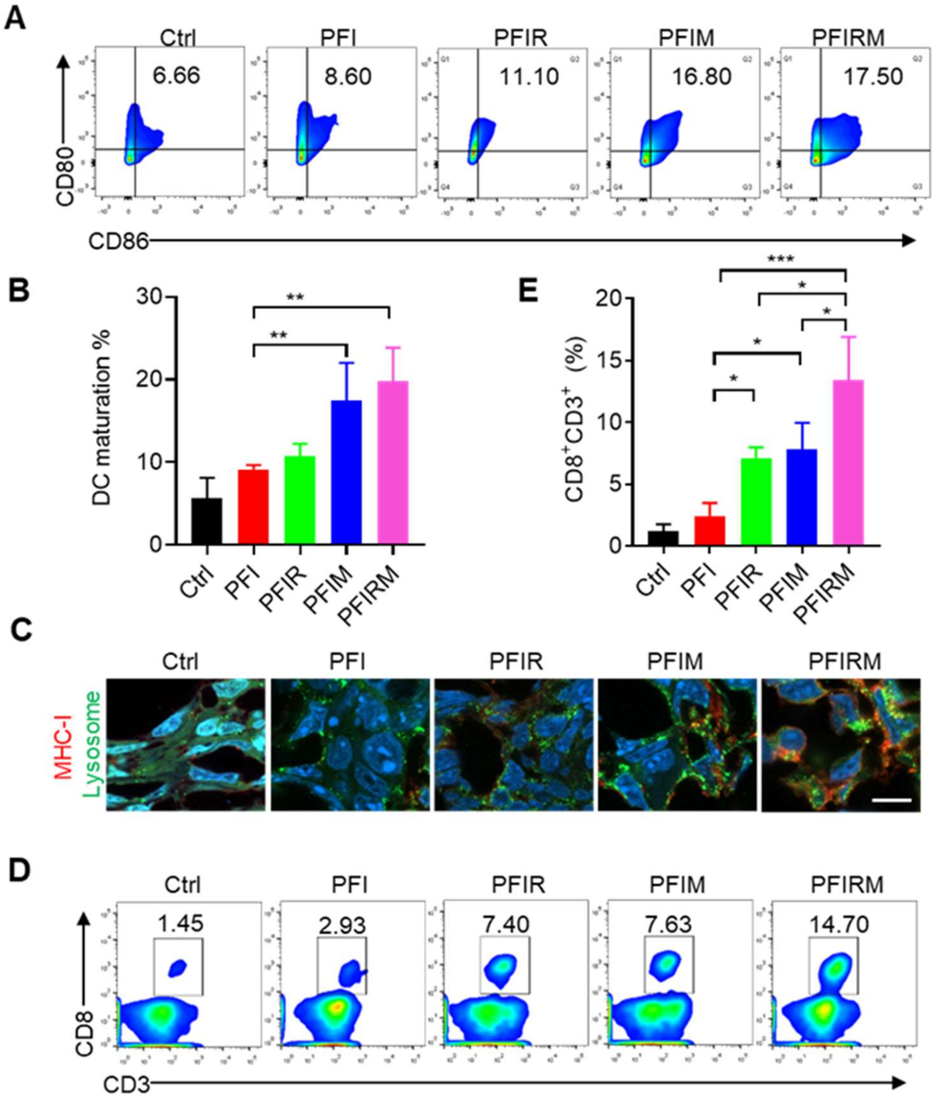

Figure S21. Mechanism validation of photothermal therapy of intramuscular tumors. (A–B) Flow cytometry analysis of the percentage of $CD80^+CD86^+$ DCs in draining lymph nodes (n=3). (C) Immunofluorescent staining of MHC-I and LAMP2. Scale bar is 10 μm. (D-E) Flow cytometry analysis of the percent of $CD8^+CD3^+$ T cells in tumors (n=3). Data represent mean ± SD. *$P < 0.05$, **$P < 0.01$ and ***$P < 0.001$.

Reference

1. Luo G, Sun Y, Zhang J, Xu Z, Lu W, Wang H, et al. Nanodefensin-encased hydrogel with dual bactericidal and pro-regenerative functions for advanced wound therapy. Theranostics. 2021; 11: 3642-60.
2. Shimoyama M, Tatsuoka H, Ohtori S, Tanaka K, Shimoyama N. Change of dorsal horn neurochemistry in a mouse model of neuropathic cancer pain. Pain. 2005; 114: 221-30.
3. Wang X, Zhou C, Liang P, Yang J, Li F, Liao D, et al. Characterization of Specific Roles of Sodium Channel Subtypes in Regional Anesthesia. Reg Anesth Pain Med. 2015; 40: 599-604.
4. Younis MR, Wang C, An R, Wang S, Younis MA, Li ZQ, et al. Low Power Single Laser Activated Synergistic Cancer Phototherapy Using Photosensitizer Functionalized Dual Plasmonic Photothermal Nanoagents. ACS Nano. 2019; 13: 2544-57.
5. Liu C, Zhang S, Li J, Wei J, Mullen K, Yin M. A Water-Soluble, NIR-Absorbing Quaterrylenediimide Chromophore for Photoacoustic Imaging and Efficient Photothermal Cancer Therapy. Angew Chem Int Ed Engl. 2019; 58: 1638-42.
